# Supplementary material for: Virtual 2D map of cyanobacterial proteomes
Source: PLoS One. 2022 Oct 3;17(10):e0275148. doi: 10.1371/journal.pone.0275148 (PMC9529120; doi:10.1371/journal.pone.0275148)
Supplement: S1 Fig — (PPTX) [file pone.0275148.s001.pptx]

## Slide 1
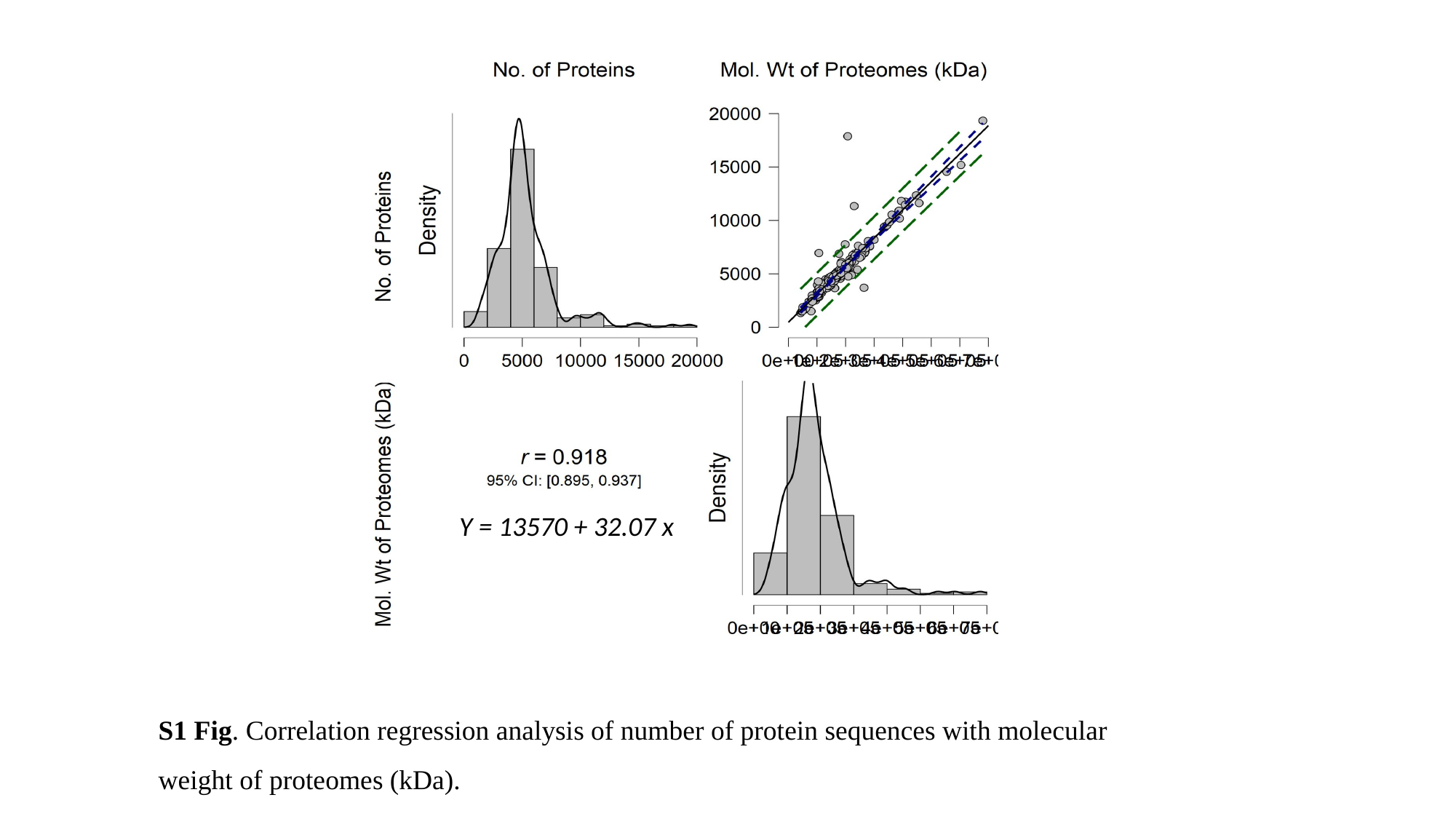

Y = 13570 + 32.07 x
S1 Fig. Correlation regression analysis of number of protein sequences with molecular weight of proteomes (kDa).
