# Supplementary figures and images for: Virtual 2D map of cyanobacterial proteomes
Source: PLoS One. 2022 Oct 3;17(10):e0275148. doi: 10.1371/journal.pone.0275148 (PMC9529120; doi:10.1371/journal.pone.0275148)

## Slide 1
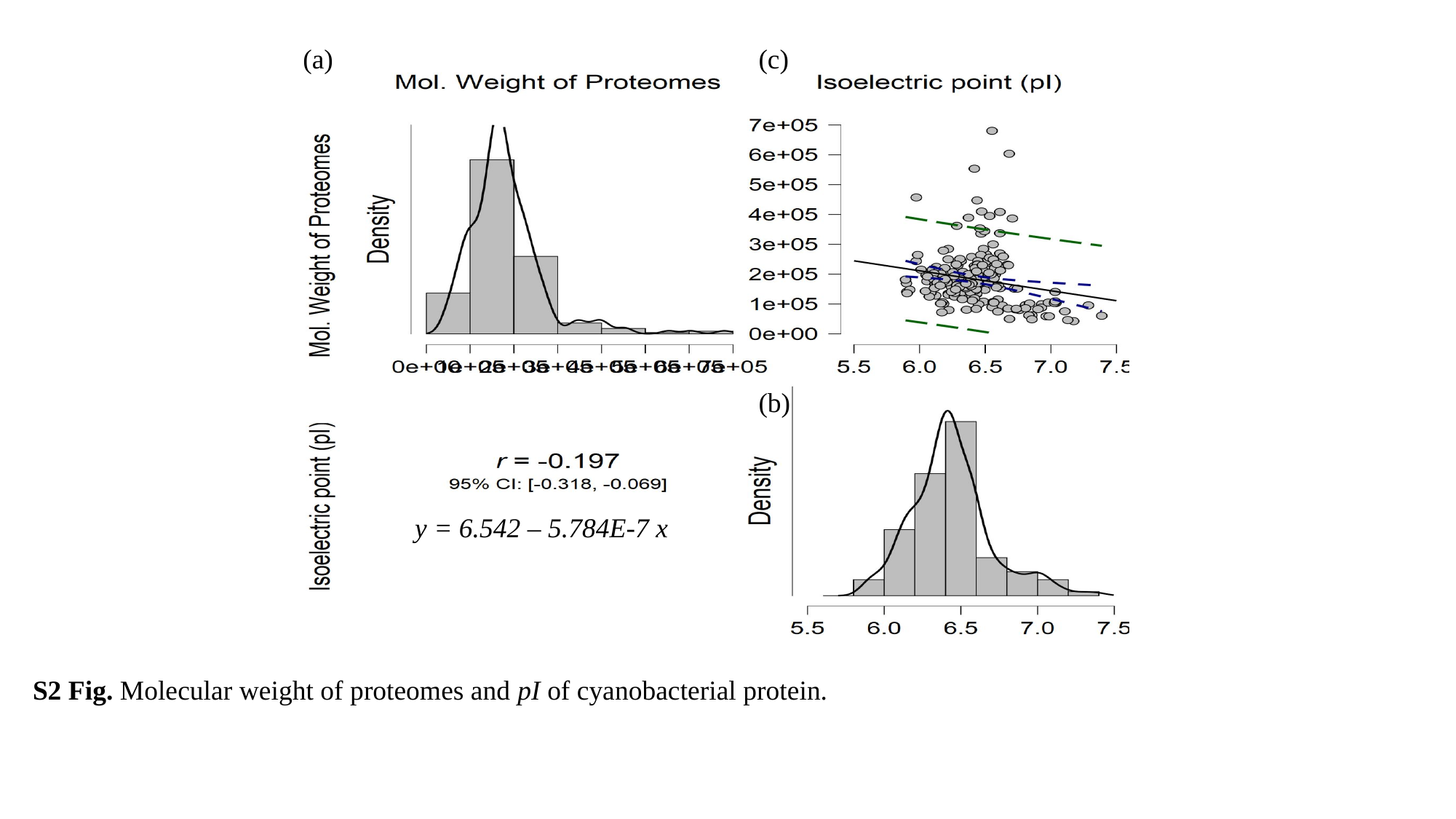

(a)
(c)
y = 6.542 – 5.784E-7 x
(b)
S2 Fig. Molecular weight of proteomes and pI of cyanobacterial protein.

Supplement: S2 Fig — (PPTX) [file pone.0275148.s002.pptx]

## Slide 1
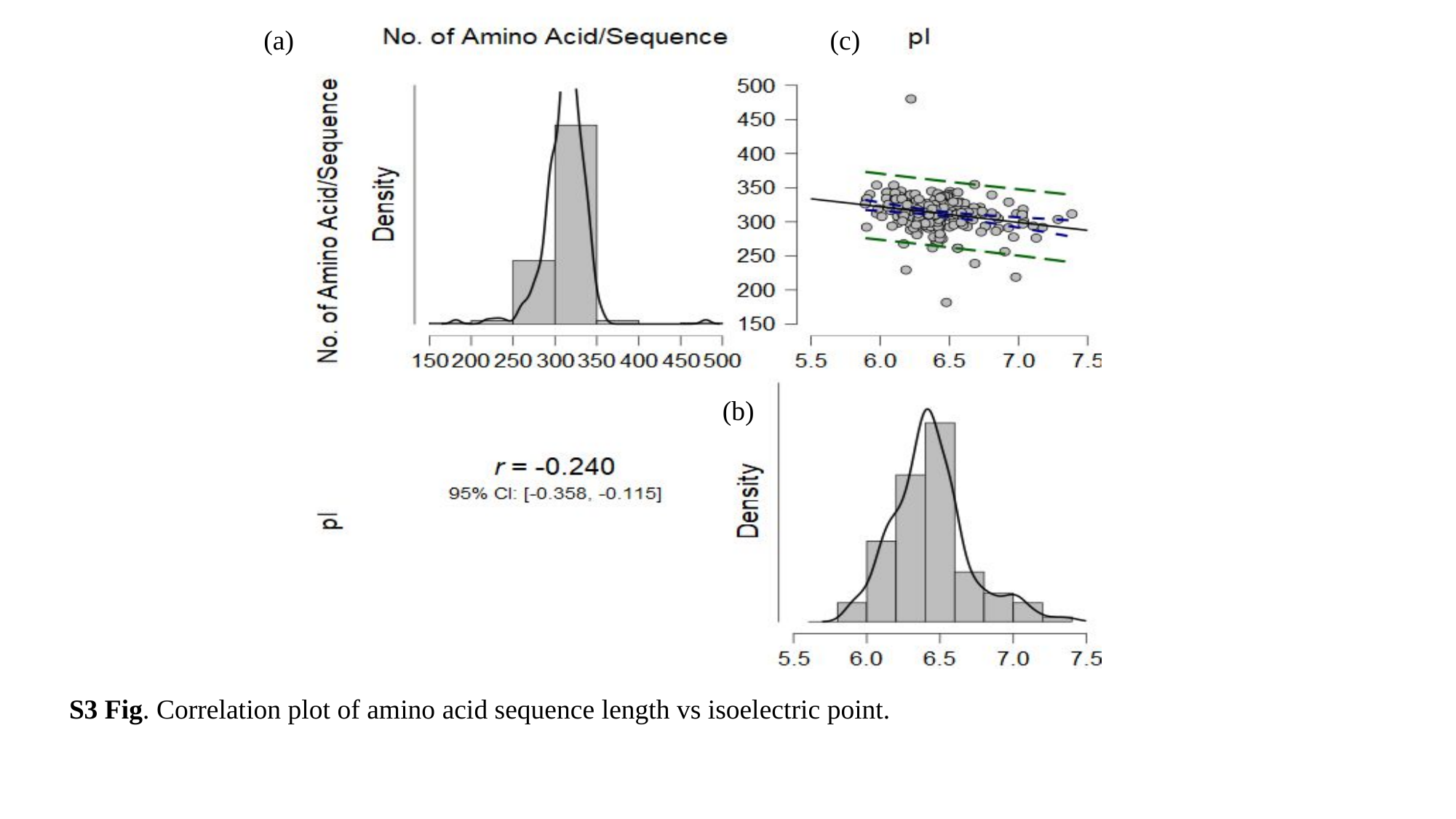

(a)
(c)
(b)
S3 Fig. Correlation plot of amino acid sequence length vs isoelectric point.

Supplement: S3 Fig — (PPTX) [file pone.0275148.s003.pptx]

## Slide 1
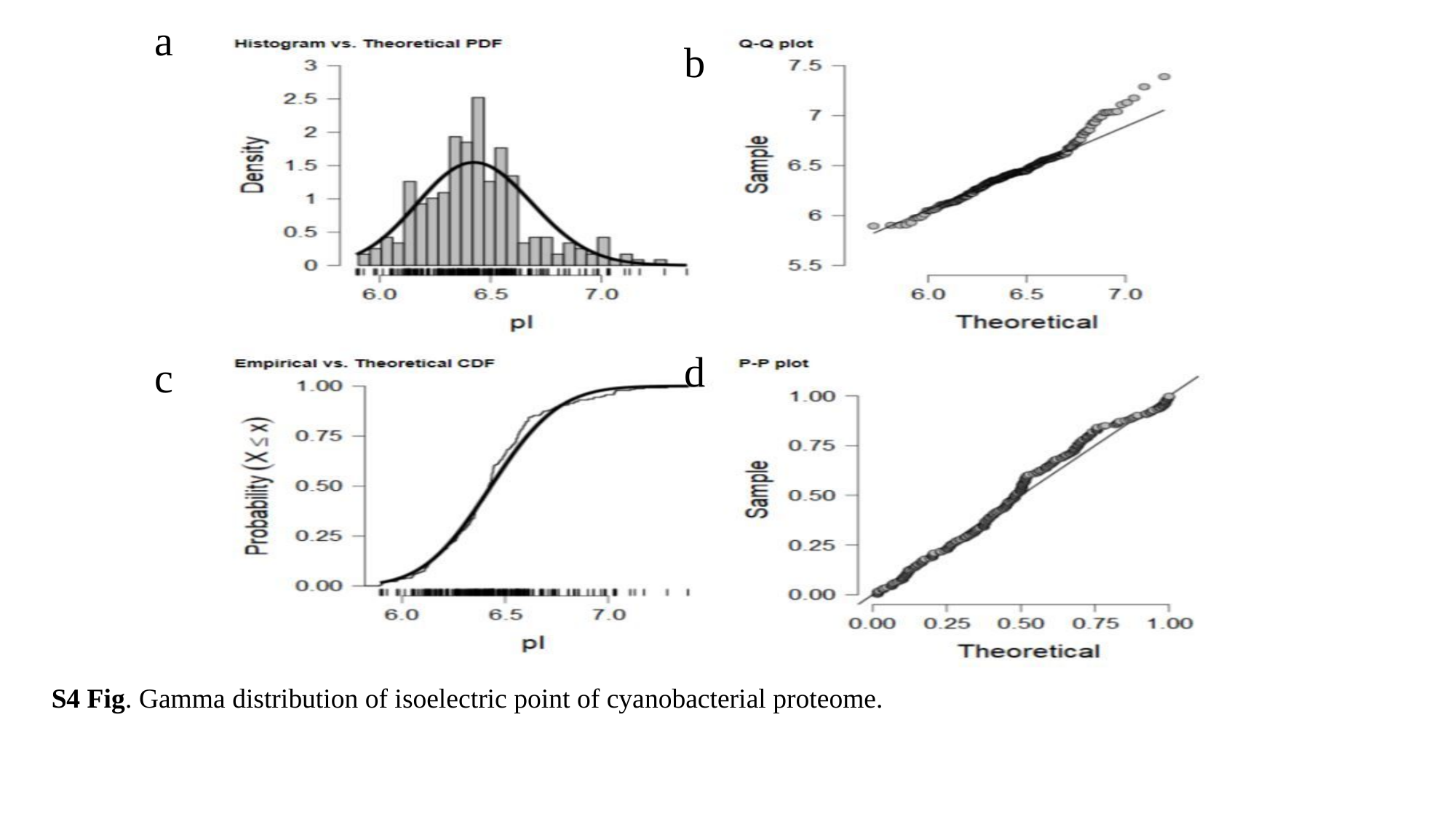

a
b
d
c
S4 Fig. Gamma distribution of isoelectric point of cyanobacterial proteome.

Supplement: S4 Fig — (PPTX) [file pone.0275148.s004.pptx]

## Slide 1
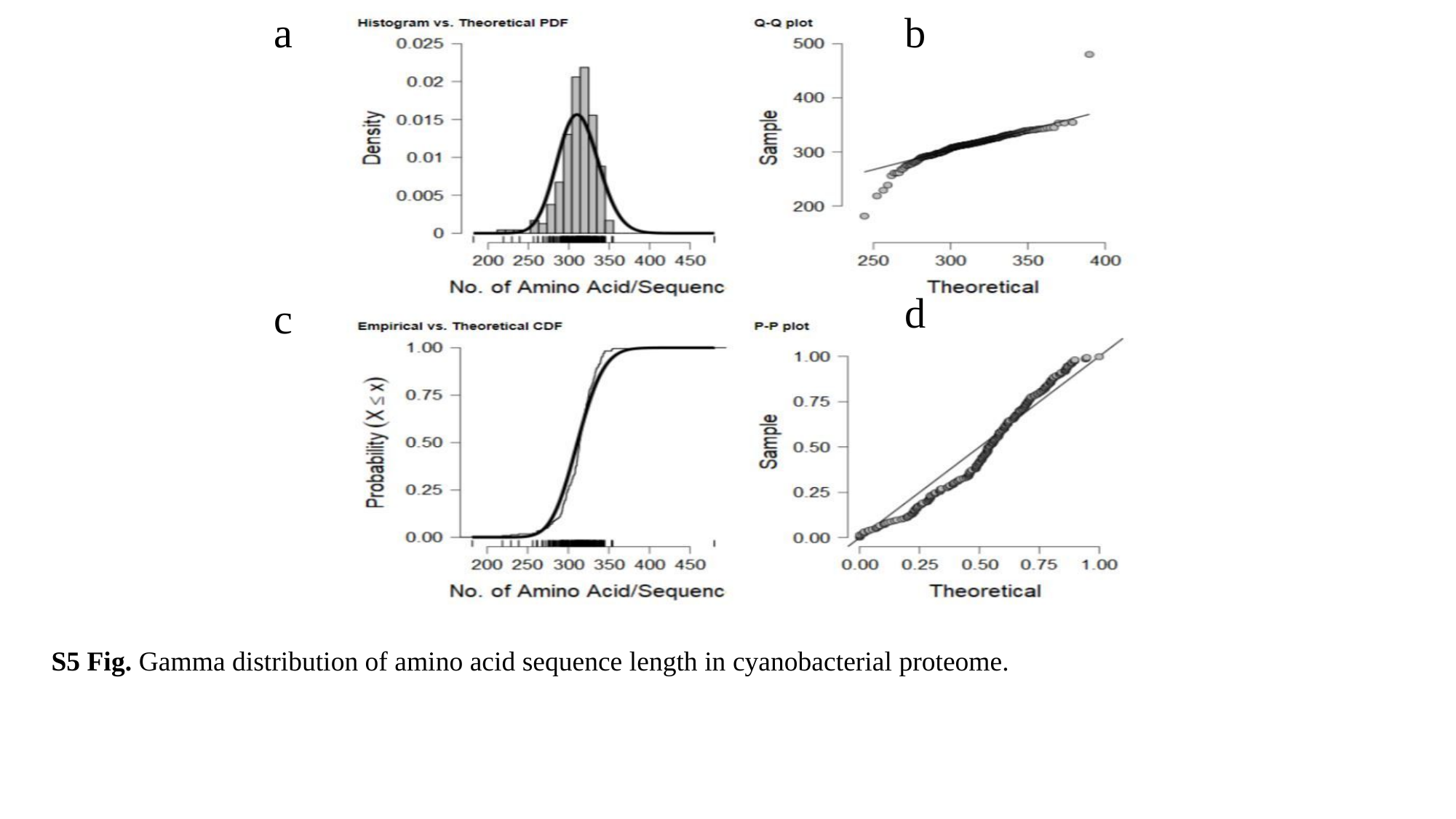

a
b
d
c
S5 Fig. Gamma distribution of amino acid sequence length in cyanobacterial proteome.

Supplement: S5 Fig — (PPTX) [file pone.0275148.s005.pptx]
