# Supplementary material for: Virtual 2D map of cyanobacterial proteomes
Source: PLoS One. 2022 Oct 3;17(10):e0275148. doi: 10.1371/journal.pone.0275148 (PMC9529120; doi:10.1371/journal.pone.0275148)
Supplement: S1 Table — (DOCX) [file pone.0275148.s006.docx]

**S1 Table.** Table depicting the list of the cyanobacterial species with the number of protein sequences used in this study.

| **Species name** | **Evolutionary Time Scale (MYA)** | **Order** | **No. of Protein sequences** | **Mol. wt of the proteome (kDa)** | **Average Weight of protein** | **Average *pI*** |
| --- | --- | --- | --- | --- | --- | --- |
| *Acaryochloris marina* | 687 | [Synechococcales](https://www.ncbi.nlm.nih.gov/Taxonomy/Browser/wwwtax.cgi?mode=Undef&id=1890424&lvl=3&p=has_linkout&p=blast_url&p=genome_blast&lin=f&keep=1&srchmode=1&unlock) | 6998 | 243885.2612 | 34.8507 | 6.316 |
| *Acaryochloris* sp. CCMEE5410 | 687 | [Synechococcales](https://www.ncbi.nlm.nih.gov/Taxonomy/Browser/wwwtax.cgi?mode=Undef&id=1890424&lvl=3&p=has_linkout&p=blast_url&p=genome_blast&lin=f&keep=1&srchmode=1&unlock) | 6669 | 228968.4655 | 34.3332 | 6.291 |
| *Acaryochloris* sp. RCC1774 | 687 | [Synechococcales](https://www.ncbi.nlm.nih.gov/Taxonomy/Browser/wwwtax.cgi?mode=Undef&id=1890424&lvl=3&p=has_linkout&p=blast_url&p=genome_blast&lin=f&keep=1&srchmode=1&unlock) | 5307 | 183168.6185 | 34.5145 | 6.223 |
| *Aliterella atlantica* | NA | [Chroococcidiopsidales](https://www.ncbi.nlm.nih.gov/Taxonomy/Browser/wwwtax.cgi?mode=Undef&id=1890505&lvl=3&lin=f&keep=1&srchmode=1&unlock) | 4756 | 163374.5717 | 34.3512 | 6.474 |
| *Alkalinema* sp. CACIAM70d | 2140 | [Synechococcales](https://www.ncbi.nlm.nih.gov/Taxonomy/Browser/wwwtax.cgi?mode=Undef&id=1890424&lvl=3&p=has_linkout&p=blast_url&p=genome_blast&lin=f&keep=1&srchmode=1&unlock) | 5216 | 189321.1049 | 36.2962 | 6.337 |
| *Anabaena cylindrica* | 801 | Nostocales | 17878 | 207570.6568 | 35.8622 | 6.330 |
| *Anabaena* sp. 4-3 | 801 | Nostocales | 4513 | 162175.7077 | 35.9352 | 6.441 |
| *Anabaena* sp. 90 | 801 | Nostocales | 3988 | 156537.0961 | 34.8402 | 6.358 |
| *Anabaena* sp. AL09 | 801 | Nostocales | 4493 | 130447.3035 | 32.7099 | 6.333 |
| *Anabaena* sp. AL93 | 801 | Nostocales | 4693 | 152344.6364 | 32.4621 | 6.350 |
| *Anabaena* sp. CRKS33 | 801 | Nostocales | 4638 | 141379.7941 | 30.4829 | 6.401 |
| *Anabaena* sp. LE011-02 | 801 | Nostocales | 4072 | 135632.787 | 33.3086 | 6.309 |
| *Anabaena* sp. MDT14b | 801 | Nostocales | 4546 | 181355.0627 | 39.8933 | 6.419 |
| *Anabaena* sp. PCC7108 | 801 | Nostocales | 4916 | 174238.8199 | 35.4071 | 6.364 |
| *Anabaena* sp. UBA12330 | 801 | Nostocales | 3675 | 128652.8827 | 35.0075 | 6.383 |
| *Anabaena* sp. WA102 | 801 | Nostocales | 4857 | 160161.5359 | 32.9754 | 6.334 |
| *Anabaena* sp. WA113 | 801 | Nostocales | 4002 | 133183.8755 | 33.2793 | 6.435 |
| *Anabaenopsis circularis* | 801 | Nostocales | 5642 | 208069.8764 | 36.8787 | 6.430 |
| *Aphanizomenonaceae* | 36 | Nostocales | 2575 | 89496.82287 | 34.7560 | 6.553 |
| *Aphanizomenon flos-aquae* | 36 | Nostocales | 4156 | 121759.3144 | 29.2972 | 6.378 |
| *Aphanocapsa feldmannii* | 687 | [Synechococcales](https://www.ncbi.nlm.nih.gov/Taxonomy/Browser/wwwtax.cgi?mode=Undef&id=1890424&lvl=3&p=has_linkout&p=blast_url&p=genome_blast&lin=f&keep=1&srchmode=1&unlock) | 1959 | 59111.12817 | 30.1741 | 6.965 |
| *Aphanocapsa montana* | 687 | [Synechococcales](https://www.ncbi.nlm.nih.gov/Taxonomy/Browser/wwwtax.cgi?mode=Undef&id=1890424&lvl=3&p=has_linkout&p=blast_url&p=genome_blast&lin=f&keep=1&srchmode=1&unlock) | 7045 | 243650.7268 | 34.5849 | 5.974 |
| *Aphanothece* *minutissima* | 792 | [Chroococcales](https://www.ncbi.nlm.nih.gov/Taxonomy/Browser/wwwtax.cgi?mode=Undef&id=1118&lvl=3&lin=f&keep=1&srchmode=1&unlock) | 3091 | 103739.2401 | 33.5617 | 7.028 |
| *Aphanothece hegewaldii* | 792 | [Chroococcales](https://www.ncbi.nlm.nih.gov/Taxonomy/Browser/wwwtax.cgi?mode=Undef&id=1118&lvl=3&lin=f&keep=1&srchmode=1&unlock) | 4768 | 170148.3059 | 35.6854 | 6.322 |
| *Aphanothece sacrum* | 792 | [Chroococcales](https://www.ncbi.nlm.nih.gov/Taxonomy/Browser/wwwtax.cgi?mode=Undef&id=1118&lvl=3&lin=f&keep=1&srchmode=1&unlock) | 4175 | 135052.0955 | 32.3478 | 6.220 |
| *Arthrospira maxima* | 54 | [Oscillatoriales](https://www.ncbi.nlm.nih.gov/Taxonomy/Browser/wwwtax.cgi?mode=Undef&id=1150&lvl=3&lin=f&keep=1&srchmode=1&unlock) | 4867 | 168104.8484 | 34.5397 | 6.350 |
| *Arthrospira* | 54 | [Oscillatoriales](https://www.ncbi.nlm.nih.gov/Taxonomy/Browser/wwwtax.cgi?mode=Undef&id=1150&lvl=3&lin=f&keep=1&srchmode=1&unlock) | 4867 | 168104.8484 | 34.5397 | 6.350 |
| *Arthrospira platensis* | 54 | [Oscillatoriales](https://www.ncbi.nlm.nih.gov/Taxonomy/Browser/wwwtax.cgi?mode=Undef&id=1150&lvl=3&lin=f&keep=1&srchmode=1&unlock) | 5162 | 172787.4936 | 33.4729 | 6.437 |
| *Aulosira laxa* | 51 | Nostocales | 7088 | 268933.3868 | 37.9420 | 6.491 |
| *Calothrix* 7103 | 1256 | Nostocales | 9350 | 337228.272 | 36.2923 | 6.612 |
| *Calothrix brevissima* | 1256 | Nostocales | 6929 | 265787.7926 | 38.3587 | 6.505 |
| *Calothrix desertica* | 1256 | Nostocales | 19335 | 680331.825 | 35.1865 | 6.552 |
| *Calothrix elsteri* | 1256 | Nostocales | 5470 | 187628.8935 | 34.3014 | 6.507 |
| *Calothrix parasitica* | 1256 | Nostocales | 6996 | 266808.1901 | 38.1372 | 6.506 |
| *Calothrix rhizosoleniae* | 1256 | Nostocales | 4602 | 162407.8094 | 35.2907 | 6.593 |
| *Calothrix* sp. PCC7507 | 1256 | Nostocales | 11752 | 410028.4304 | 34.8900 | 6.473 |
| *Candidate division* WOR-1 | 1426-2635 | Melainabacteria | 2800 | 95538.3774 | 34.1208 | 7.287 |
| *Candidatus gastranaerophilales* | 1426-2635 | Melainabacteria | 1818 | 62367.94141 | 34.3057 | 6.855 |
| *Candidatus margulisbacteria* | 1426-2635 | Melainabacteria | 2534 | 96612.75946 | 38.1265 | 6.808 |
| *Candidatus marinamargulisbacteria* | 1426-2635 | Melainabacteria | 1683 | 58856.397 | 34.9711 | 6.988 |
| *Candidatus melainabacteria* | 1426-2635 | Melainabacteria | 2741 | 99458.08178 | 36.2853 | 6.930 |
| *Candidatus gaganbacteria* | 1426-2635 | Melainabacteria | 1751 | 60631.75099 | 34.6269 | 7.388 |
| *Candidatus synechococcus spongiarum* | 1426-2635 | Melainabacteria | 1337 | 42726.76694 | 31.9571 | 7.174 |
| *Candidatus termititenax* | 1426-2635 | Melainabacteria | 2309 | 75610.22504 | 32.7458 | 7.107 |
| *Chamaesiphon minutus* | 792 | Synechococcales | 11824 | 394990.4121 | 33.4058 | 6.534 |
| *Chamaesiphon polymorphus* | 792 | Synechococcales | 5197 | 171994.203 | 33.0949 | 6.413 |
| *Chlorogloeopsis fritschii* | 1871 | Nostocales | 6541 | 228343.8082 | 34.9096 | 6.541 |
| *Chondrocystis* sp. NIES-4102 | 1871 | Chroococcales | 4666 | 159344.2738 | 34.1500 | 6.353 |
| *Chroococcales cyanobacterium* | 1862 | Chroococcales | 5044 | 177686.9187 | 35.2273 | 6.370 |
| *Chroococcidiopsis cubana* | 1862 | Chroococcidiopsidales | 6893 | 232174.171 | 33.6826 | 6.669 |
| *Chroococcidiopsis thermalis* | 1862 | Chroococcidiopsidales | 11462 | 408502.6867 | 35.6397 | 6.612 |
| *Chroogloeocystis siderophila* | 1871 | Chroococcales | 4219 | 147503.8365 | 34.9618 | 6.497 |
| *Chrysosporum ovalisporum* | 1862 | Nostocales | 2851 | 107851.6588 | 37.8294 | 6.488 |
| *Coleofasciculus chthonoplastes* | 1301 | [Oscillatoriales](https://www.ncbi.nlm.nih.gov/Taxonomy/Browser/wwwtax.cgi?mode=Undef&id=1150&lvl=3&lin=f&keep=1&srchmode=1&unlock) | 6596 | 249974.3735 | 37.8978 | 6.218 |
| *Crinalium epipsammum* | 1301 | [Oscillatoriales](https://www.ncbi.nlm.nih.gov/Taxonomy/Browser/wwwtax.cgi?mode=Undef&id=1150&lvl=3&lin=f&keep=1&srchmode=1&unlock) | 9426 | 335899.9355 | 35.6354 | 6.468 |
| *Crocosphaera watsonii* | 1241 | Chroococcales | 4840 | 154468.0831 | 31.9148 | 6.268 |
| *Cuspidothrix issatschenkoi* | 91 | Nostocales | 3780 | 131881.9668 | 34.8894 | 6.362 |
| *Cyanobacteria* 5 | 1241 | Melainabacteria | 5622 | 197773.5588 | 35.1785 | 6.576 |
| *Cyanobacteria* | 1241 | Melainabacteria | 6998 | 243885.2612 | 34.8507 | 6.316 |
| *Cyanobacterium aponinum* | 1241 | Chroococcales | 6847 | 250936.0838 | 36.6490 | 6.308 |
| *Cyanobacterium* PCC 7702 | 1241 | Chroococcales | 3962 | 140695.875 | 35.5113 | 7.033 |
| *Cyanobacterium stanieri* PCC 7202 | 1241 | Chroococcales | 3962 | 101486.9486 | 35.7726 | 6.184 |
| *Cyanobacterium* SU3 | 1241 | Chroococcales | 5102 | 169796.4686 | 33.2803 | 6.261 |
| *Cyanobium gracile* | 108 | [Synechococcales](https://www.ncbi.nlm.nih.gov/Taxonomy/Browser/wwwtax.cgi?mode=Undef&id=1890424&lvl=3&p=has_linkout&p=blast_url&p=genome_blast&lin=f&keep=1&srchmode=1&unlock) | 3176 | 103375.49 | 32.5489 | 7.039 |
| *Cyanobium* sp. | 108 | [Synechococcales](https://www.ncbi.nlm.nih.gov/Taxonomy/Browser/wwwtax.cgi?mode=Undef&id=1890424&lvl=3&p=has_linkout&p=blast_url&p=genome_blast&lin=f&keep=1&srchmode=1&unlock) | 2786 | 87877.79556 | 31.5426 | 6.929 |
| *Cyanobium usitatum* | 108 | [Synechococcales](https://www.ncbi.nlm.nih.gov/Taxonomy/Browser/wwwtax.cgi?mode=Undef&id=1890424&lvl=3&p=has_linkout&p=blast_url&p=genome_blast&lin=f&keep=1&srchmode=1&unlock) | 2526 | 80216.8955 | 31.7564 | 6.759 |
| *Cyanosarcina burmensis* |  | Chroococcales | 6334 | 224934.278 | 35.5122 | 6.600 |
| *Cyanothece* sp. | 242 | [Oscillatoriales](https://www.ncbi.nlm.nih.gov/Taxonomy/Browser/wwwtax.cgi?mode=Undef&id=1150&lvl=3&lin=f&keep=1&srchmode=1&unlock) | 10543 | 361997.5905 | 34.3353 | 6.284 |
| *Cylindrospermopsis raciborskii* | 91 | Nostocales | 3200 | 115350.3198 | 36.0469 | 6.597 |
| *Cylindrospermum stagnale* | 702 | Nostocales | 12358 | 447359.8407 | 36.2000 | 6.438 |
| *Dactylococcopsis salina* | 600 | [Synechococcales](https://www.ncbi.nlm.nih.gov/Taxonomy/Browser/wwwtax.cgi?mode=Undef&id=1890424&lvl=3&p=has_linkout&p=blast_url&p=genome_blast&lin=f&keep=1&srchmode=1&unlock) | 6749 | 224478.8124 | 33.2610 | 6.128 |
| *Desertifilum* sp. IPPASB-1220 | 72 | [Oscillatoriales](https://www.ncbi.nlm.nih.gov/Taxonomy/Browser/wwwtax.cgi?mode=Undef&id=1150&lvl=3&lin=f&keep=1&srchmode=1&unlock) | 4976 | 190797.9578 | 38.3436 | 6.151 |
| *Dolichospermum circinale* | 91 | Nostocales | 3759 | 126641.0252 | 33.6900 | 6.399 |
| *Dolichospermum compactum* | 91 | Nostocales | 4227 | 142183.3907 | 33.6369 | 6.341 |
| *Euhalothece* sp. KZN001 | 600 | Chroococcales | 4332 | 140690.4747 | 32.4770 | 5.902 |
| Filamentouscyanobacterium ESFC-1 | 1241 | [Oscillatoriales](https://www.ncbi.nlm.nih.gov/Taxonomy/Browser/wwwtax.cgi?mode=Undef&id=1150&lvl=3&lin=f&keep=1&srchmode=1&unlock) | 4802 | 172506.2273 | 35.9238 | 6.123 |
| Filamentouscyanobacterium Phorm 46 | 1241 | [Oscillatoriales](https://www.ncbi.nlm.nih.gov/Taxonomy/Browser/wwwtax.cgi?mode=Undef&id=1150&lvl=3&lin=f&keep=1&srchmode=1&unlock) | 4293 | 140397.4133 | 32.7037 | 6.328 |
| *Fischerella major* | 1590 | Nostocales | 4501 | 162762.9358 | 36.1615 | 6.584 |
| *Fischerella muscicola* CCMEE5323 | 1590 | Nostocales | 5325 | 186145.7811 | 34.9569 | 6.557 |
| *Fischerella* sp. PCC9431 | 1590 | Nostocales | 5737 | 207227.9761 | 36.1213 | 6.568 |
| *Fischerella thermalis* | 1590 | Nostocales | 4271 | 153058.4136 | 35.8366 | 6.613 |
| *Fortiea contorta* | 1590 | Nostocales | 4916 | 169216.348 | 34.4215 | 6.522 |
| *Fremyella diplosiphon* | 1590 | Nostocales | 7571 | 284723.1652 | 37.6070 | 6.487 |
| *Geitlerinema* sp. PCC7105 | 792 | Oscillatoriales | 4755 | 175154.9745 | 36.8359 | 6.185 |
| *Geitlerinema* sp. PCC7407 | 792 | Oscillatoriales | 7602 | 284730.1327 | 37.4546 | 6.219 |
| *Geminocystis herdmanii* | 1376 | Chroococcales | 3906 | 130942.3266 | 33.5233 | 6.219 |
| *Gloeobacter kilaueensis* | 1341 | Gloeobacterales | 4302 | 151550.8451 | 35.2279 | 6.723 |
| *Gloeobacter violaceus* | 1341 | Gloeobacterales | 4430 | 151629.3678 | 34.2278 | 6.745 |
| *Gloeocapsa* sp. PCC73106 | 1341 | Chroococcales | 3723 | 128140.3325 | 34.4185 | 6.124 |
| *Gloeocapsopsis* sp. AAB1 | 1341 | Chroococcales | 4747 | 161491.4276 | 34.0196 | 6.441 |
| *Gloeomargarita lithophora* | 2245 | Gloeoemargaritales | 2930 | 95144.84444 | 32.4726 | 6.630 |
| *Halomicronema excentricum* | 452 | Synechococcales | 4628 | 169405.924 | 36.6045 | 5.900 |
| *Halomicronema hongdechloris* | 452 | Synechococcales | 4641 | 157473.5619 | 33.9309 | 6.242 |
| *Halothece* sp. PCC7418 | 2180 | Chroococcales | 3703 | 264542.7463 | 35.6574 | 5.986 |
| *Hapalosiphonaceae cyanobacterium* | 57 | Nostocales | 5625 | 199953.2259 | 35.5472 | 6.553 |
| *Hapalosiphon aceae* | 57 | Nostocales | 6275 | 231057.3613 | 36.8218 | 6.576 |
| *Hapalosiphon* sp. MRB220 | 57 | Nostocales | 5927 | 214328.8505 | 36.1614 | 6.548 |
| *Hassallia byssoidea* | 2180 | Nostocales | 10909 | 386737.6332 | 35.4512 | 6.707 |
| *Hydrococcus rivularis* | 2180 | [Pleurocapsales](https://www.ncbi.nlm.nih.gov/Taxonomy/Browser/wwwtax.cgi?mode=Undef&id=52604&lvl=3&keep=1&srchmode=1&unlock) | 4381 | 157670.2425 | 35.9895 | 6.588 |
| *Hydrocoleum* sp. CS-953 | 1301 | Oscillatoriales | 5237 | 171735.6965 | 32.7927 | 6.368 |
| *Kamptonema formosum* | 1474 | Oscillatoriales | 5464 | 206792.8294 | 37.8464 | 6.253 |
| *Leptolyngbya antarctica* | 1539 | Synechococcales | 4267 | 137488.4337 | 32.2213 | 6.086 |
| *Leptolyngbya boryana* | 1539 | Synechococcales | 6512 | 227869.0892 | 34.9921 | 6.419 |
| *Leptolyngbya foveolarum* | 1539 | Synechococcales | 4289 | 143269.4565 | 33.4039 | 6.271 |
| *Leptolyngbya frigida* | 1539 | Synechococcales | 5894 | 204148.3199 | 34.6366 | 6.512 |
| *Leptolyngbya ohadii* | 1539 | Synechococcales | 6302 | 232754.7309 | 36.9334 | 6.280 |
| *Leptolyngbya sphensonii* | 1539 | Synechococcales | 5045 | 164548.0071 | 33.7188 | 6.434 |
| *Leptolyngbya* sp. JSC-1 | 1539 | Synechococcales | 6109 | 185637.8586 | 36.7964 | 6.404 |
| *Leptolyngbya* sp. | 1539 | Synechococcales | 4880 | 220694.3697 | 36.1261 | 6.193 |
| *Leptolyngbya valderiana* | 1539 | Synechococcales | 5553 | 215507.4697 | 38.8091 | 6.099 |
| *Limnoraphis robusta* | 616 | Oscillatoriales | 5269 | 197314.3105 | 37.4481 | 6.135 |
| *Limnothrix rosea* | 1560 | Synechococcales | 3540 | 124816.4788 | 35.2588 | 6.075 |
| *Limnothrix* sp. CACIAM69d | 1560 | Synechococcales | 3718 | 134428.9724 | 36.1562 | 6.435 |
| *Lyngbya* 3 | 1539 | Oscillatoriales | 5136 | 196996.551 | 38.3560 | 6.050 |
| *Lyngbya aestuarii* | 1539 | Oscillatoriales | 5136 | 196996.551 | 38.3560 | 6.050 |
| *Lyngbya confervoides* | 1539 | Oscillatoriales | 6348 | 215163.7078 | 33.8947 | 6.012 |
| *Lyngbya* sp. PCC8106 | 1539 | Oscillatoriales | 5548 | 209693.2396 | 37.7961 | 6.142 |
| *Mastigocladopsis repens* | 777 | Nostocales | 5283 | 186076.1006 | 35.2216 | 6.568 |
| *Mastigocladus laminosus* | 777 | Nostocales | 5531 | 199052.8693 | 35.9885 | 6.544 |
| *Mastigocoleus testarum* | 777 | Nostocales | 15190 | 603575.5146 | 39.7350 | 6.683 |
| *Merismopedia glauca* | 1437 | Synechococcales | 4559 | 157860.2653 | 34.6260 | 6.404 |
| *Microcoleaceae bacterium* | 1753 | Oscillatoriales | 4366 | 144410.9888 | 33.0762 | 6.406 |
| *Microcoleus* sp. PCC7113 | 1753 | Oscillatoriales | 1500 | 79976.05187 | 53.3173 | 6.223 |
| *Microcoleus vaginatus* | 1753 | Oscillatoriales | 10187 | 389265.5416 | 38.2119 | 6.373 |
| *Microcystis aeruginosa* | 1437 | Chroococcales | 4762 | 149653.3603 | 31.4265 | 6.432 |
| *Microcystis flos-aquae* | 1437 | Chroococcales | 4486 | 145452.0934 | 32.4235 | 6.424 |
| *Microcystis panniformis* | 1437 | Chroococcales | 4476 | 139427.6086 | 31.1500 | 6.387 |
| *Microcystis* sp. 0824 | 1437 | Chroococcales | 3641 | 119234.3209 | 32.7476 | 6.419 |
| *Microcystis viridis* | 1437 | Chroococcales | 4762 | 149653.3603 | 31.4265 | 6.432 |
| *Moorea bouillonii* | 1753 | Oscillatoriales | 5996 | 217947.1774 | 36.3487 | 6.500 |
| *Moorea producens* | 1753 | Oscillatoriales | 6221 | 219943.2407 | 35.3549 | 6.572 |
| *Myxosarcina* sp. GI1 | 1898 | Pleurocapsales | 5986 | 211204.1083 | 35.2830 | 6.486 |
| *Neosynechococcus sphagnicola* | 1618 | Synechococcales | 3256 | 99144.37536 | 30.4497 | 6.450 |
| *Nodosilinea nodulosa* | 2032 | Synechococcales | 5911 | 201668.9734 | 34.1175 | 6.163 |
| *Nodularia* sp. NIES-3585 | 1040 | Nostocales | 4739 | 166737.2904 | 35.1840 | 6.295 |
| *Nodularia spumigena 2* | 1040 | Nostocales | 4509 | 158740.6526 | 35.2052 | 6.362 |
| *Nodularia spumigena* 3 | 1040 | Nostocales | 4431 | 152238.9002 | 35.0216 | 6.352 |
| *Nodularia spumigena* | 1040 | Nostocales | 4347 | 156409.9664 | 35.2990 | 6.349 |
| Nostocaceae | 1040 | Nostocales | 5410 | 179999.0441 | 33.2715 | 6.415 |
| *Nostocales cyanobacterium* | 1040 | Nostocales | 4759 | 209432.1637 | 37.3719 | 6.434 |
| Nostocales | 1040 | Nostocales | 3661 | 106734.341 | 29.1544 | 6.561 |
| *Nostoc azollae* 0708 | 1040 | Nostocales | 3661 | 162520.6696 | 34.1501 | 6.283 |
| *Nostoc calcicola* | 1040 | Nostocales | 6952 | 106734.341 | 29.1544 | 6.561 |
| *Nostoc carneum* | 1040 | Nostocales | 7034 | 257840.03 | 37.0886 | 6.397 |
| *Nostoc commune* | 1040 | Nostocales | 7419 | 264609.0789 | 37.6185 | 6.467 |
| *Nostoc cycadae* WK-1 | 1040 | Nostocales | 5398 | 241460.1933 | 32.5461 | 6.502 |
| *Nostoc flagelliforme* CCNUN1 | 1040 | Nostocales | 7764 | 198572.1652 | 36.7862 | 6.445 |
| *Nostoc linckia* | 1040 | Nostocales | 6616 | 255179.8991 | 32.8670 | 6.533 |
| *Nostoc minutum* | 1040 | Nostocales | 7625 | 244218.2227 | 36.9132 | 6.444 |
| *Nostoc* | 1040 | Nostocales | 5410 | 179999.0441 | 33.2715 | 6.415 |
| *Nostoc piscinale* | 1040 | Nostocales | 5202 | 175768.6384 | 33.7886 | 6.422 |
| *Nostoc punctiforme* | 1040 | Nostocales | 14555 | 553428.8732 | 38.0232 | 6.418 |
| *Nostoc sphaeroides* | 1040 | Nostocales | 5410 | 179999.0441 | 33.2715 | 6.415 |
| *Nostoc* sp. lobaria | 1040 | Nostocales | 5872 | 205068.9359 | 34.9231 | 6.410 |
| *Nostoc* sp. Peltigeramalaceacyanobiont DB3992 | 1040 | Nostocales | 5972 | 183442.8056 | 30.7171 | 6.431 |
| *Okeania hirsuta* | 1301 | Oscillatoriales | 5335 | 184657.159 | 34.6124 | 6.392 |
| *Oscillatoria acuminata* | 1157 | Oscillatoriales | 11628 | 457197.2525 | 39.3186 | 5.975 |
| *Oscillatoriales bacterium* | 1157 | Oscillatoriales | 2411 | 72173.51797 | 29.9350 | 6.170 |
| *Oscillatoriales cyanobacterium* JSC-12 | 1157 | Oscillatoriales | 9531 | 344410.9648 | 36.1358 | 6.495 |
| *Oscillatoriales cyanobacterium* | 1157 | Oscillatoriales | 5844 | 200095.7404 | 34.2395 | 6.372 |
| Oscillatoriales | 1157 | Oscillatoriales | 6336 | 232605.6889 | 36.7117 | 6.441 |
| *Oscillatoria nigro-viridis* | 1157 | Oscillatoriales | 6336 | 232605.6889 | 36.7117 | 6.441 |
| *Oscillatoria* sp. PCC10802 | 1157 | Oscillatoriales | 6519 | 230179.5817 | 35.3090 | 6.677 |
| Oscillatoriophycideae | 1157 | Oscillatoriales | 4883 | 172746.9778 | 35.3772 | 6.210 |
| *Phormidesmis priestleyi* | 2140 | [Synechococcales](https://www.ncbi.nlm.nih.gov/Taxonomy/Browser/wwwtax.cgi?mode=Undef&id=1890424&lvl=3&lin=f&keep=1&srchmode=1&unlock) | 4035 | 124926.4887 | 30.9607 | 6.266 |
| *Phormidium ambiguum* | 689 | Oscillatoriales | 6166 | 232030.1046 | 37.6305 | 6.442 |
| *Phormidium lacuna* | 689 | Oscillatoriales | 3904 | 147925.1859 | 37.8906 | 5.926 |
| *Phormidium* sp. SL48-SHIP | 689 | Oscillatoriales | 3667 | 136183.7848 | 37.1376 | 5.905 |
| *Phormidium tenue* | 689 | Oscillatoriales | 4996 | 176485.9022 | 35.3254 | 6.056 |
| *Phormidium willei* | 689 | Oscillatoriales | 3861 | 143310.9008 | 37.1175 | 6.045 |
| *Planktothricoides* sp. SR001 | 1301 | Oscillatoriales | 5455 | 193468.0905 | 35.4661 | 6.263 |
| *Planktothrix agardhii* | 1301 | Oscillatoriales | 4492 | 162678.7532 | 36.2152 | 6.131 |
| *Planktothrix mougeotii* | 1301 | Oscillatoriales | 4586 | 167181.1713 | 36.4546 | 6.119 |
| *Planktothrix* tepida | 1301 | Oscillatoriales | 5433 | 202923.9659 | 37.3502 | 6.109 |
| *Planktothrix paucivesiculata* | 1301 | Oscillatoriales | 4767 | 182900.2298 | 38.3679 | 6.109 |
| *Planktothrix prolifica* | 1301 | Oscillatoriales | 4729 | 168401.1134 | 35.6103 | 6.135 |
| *Planktothrix rubescens* | 1301 | Oscillatoriales | 4715 | 171503.762 | 36.3740 | 6.134 |
| *Planktothrix serta* | 1301 | Oscillatoriales | 5259 | 192242.3373 | 36.5549 | 6.061 |
| *Pleurocapsa* sp. PCC7319 | 626 | [Pleurocapsales](https://www.ncbi.nlm.nih.gov/Taxonomy/Browser/wwwtax.cgi?mode=Undef&id=52604&lvl=3&lin=f&keep=1&srchmode=1&unlock) | 6147 | 220594.1018 | 35.8864 | 6.424 |
| *Prochlorococcus marinus* | 1545 | Synechococcales | 1493 | 46474.77806 | 31.1284 | 7.129 |
| *Prochloron didemni* | 1898 | Synechococcales | 6883 | 177110.0732 | 25.7315 | 6.187 |
| *Prochlorothrix hollandica* | 1545 | Synechococcales | 8067 | 278964.4923 | 34.5809 | 6.182 |
| *Pseudanabaena biceps* | 1100 | Synechococcales | 4409 | 159661.329 | 36.2125 | 6.393 |
| *Pseudanabaena frigida* | 1100 | Synechococcales | 4669 | 168803.3899 | 36.1540 | 6.352 |
| *Pseudanabaena* sp. | 1100 | Synechococcales | 3880 | 137403.4739 | 35.4132 | 6.269 |
| *Pseudanabaena* sp. PCC6802 | 1100 | Synechococcales | 4903 | 169846.5408 | 34.6413 | 6.444 |
| *Raphidiopsis brookii* | 702 | Nostocales | 2575 | 89496.82287 | 34.7560 | 6.553 |
| *Raphidiopsis curvata* | 1040 | Nostocales | 2892 | 104832.9321 | 36.2492 | 6.566 |
| *Richelia intracellularis* | 1040 | Nostocales | 4292 | 104935.8434 | 24.4491 | 6.981 |
| *Richelia* sp. | 1040 | Nostocales | 1508 | 48930.82514 | 32.4474 | 6.856 |
| *Rivularia* sp. PCC7116 | 1370 | Nostocales | 6498 | 249119.5737 | 38.3378 | 6.562 |
| *Roseofilum reptotaenium* | 2180 | Oscillatoriales | 4978 | 181790.3799 | 36.5187 | 5.893 |
| *Rubidibacter lacunae* | 2180 | Chroococcales | 3281 | 111797.2065 | 34.0741 | 6.402 |
| *Scytonema hofmannii* | 247 | Nostocales | 9856 | 353586.3388 | 35.8752 | 6.460 |
| *Scytonema millei* | 247 | Nostocales | 4943 | 163226.9153 | 33.0218 | 6.600 |
| *Scytonema* sp. NIES-4073 | 247 | Nostocales | 7400 | 269602.9046 | 36.4328 | 6.612 |
| *Scytonema tolypothrichoides* | 247 | Nostocales | 6017 | 212435.3883 | 35.3058 | 6.615 |
| *Snowella* sp. | 1376 | Synechococcales | 4326 | 140988.0691 | 32.5908 | 6.243 |
| *Sphaerospermopsis kisseleviana* | 702 | Nostocales | 4730 | 157583.649 | 33.3157 | 6.305 |
| *Spirulina major* | 325 | [Spirulinales](https://www.ncbi.nlm.nih.gov/Taxonomy/Browser/wwwtax.cgi?mode=Undef&id=1890443&lvl=3&lin=f&keep=1&srchmode=1&unlock) | 4161 | 153487.7144 | 36.8872 | 6.114 |
| *Spirulina subsalsa* | 325 | [Spirulinales](https://www.ncbi.nlm.nih.gov/Taxonomy/Browser/wwwtax.cgi?mode=Undef&id=1890443&lvl=3&lin=f&keep=1&srchmode=1&unlock) | 4344 | 162048.0311 | 37.3038 | 6.158 |
| *Stanieria cyanosphaera* | 1862 | [Pleurocapsales](https://www.ncbi.nlm.nih.gov/Taxonomy/Browser/wwwtax.cgi?mode=Undef&id=52604&lvl=3&lin=f&keep=1&srchmode=1&unlock) | 4728 | 168894.4027 | 35.7221 | 6.399 |
| *Stanieria* sp. NIES-3757 | 1862 | [Pleurocapsales](https://www.ncbi.nlm.nih.gov/Taxonomy/Browser/wwwtax.cgi?mode=Undef&id=52604&lvl=3&lin=f&keep=1&srchmode=1&unlock) | 4603 | 164659.8837 | 35.7722 | 6.377 |
| Synechococcaceae | 927 | Synechococcales | 2526 | 80216.8955 | 31.7564 | 6.759 |
| *Synechococcales bacterium* | 927 | Synechococcales | 2979 | 82857.97782 | 27.8140 | 6.902 |
| *Synechococcus elongatus* | 927 | Synechococcales | 2459 | 81018.65199 | 32.9478 | 6.358 |
| *Synechococcus lacustris* | 927 | Synechococcales | 2652 | 82256.72209 | 31.0168 | 6.731 |
| *Synechococcus lividus* | 927 | Synechococcales | 2222 | 74927.92923 | 33.7209 | 6.597 |
| *Synechococcus* sp. | 927 | Synechococcales | 3094 | 106235.0456 | 34.3358 | 6.172 |
| *Synechococcus* sp. 1G10 | 927 | Synechococcales | 3273 | 101919.7571 | 31.1395 | 6.838 |
| *Synechococcus* sp. 65AY640 | 927 | Synechococcales | 2445 | 84815.99965 | 34.6895 | 6.675 |
| *Synechococcus* sp. BO8801 | 927 | Synechococcales | 3091 | 103739.2401 | 33.5617 | 7.028 |
| *Synechococcus* sp. CC9616 | 927 | Synechococcales | 2708 | 83855.33118 | 30.9657 | 6.432 |
| *Synechococcus* sp. NKBG 042902 | 927 | Synechococcales | 3045 | 101842.5136 | 33.4458 | 6.162 |
| *Synechococcus* sp. PCC 6312 | 927 | Synechococcales | 3542 | 116289.5559 | 32.9993 | 6.326 |
| *Synechocystis* sp. PCC 7509 | 927 | Synechococcales | 4684 | 155792.8045 | 33.2606 | 6.585 |
| *Thermosynechococcus elongatus* | 1871 | Synechococcales | 2476 | 85990.31672 | 34.7295 | 6.807 |
| *Thermosynechococcus vulcanus* | 1871 | Synechococcales | 2402 | 84034.4782 | 34.9852 | 6.737 |
| *Tolypothrix bouteillei* | 103 | Nostocales | 8193 | 299929.9309 | 36.6080 | 6.560 |
| *Tolypothrix campylonemoides* | 103 | Nostocales | 7414 | 259137.7764 | 34.9524 | 6.637 |
| *Tolypothrix* sp. NIES-4075 | 103 | Nostocales | 6708 | 234200.3428 | 34.9135 | 6.588 |
| *Trichodesmium erythraeum* | 616 | Oscillatoriales | 11343 | 230224.238 | 20.2965 | 6.479 |
| *Trichodesmium thiebautii* | 616 | Oscillatoriales | 1868 | 50144.32502 | 26.8438 | 6.685 |
| *Trichormus* sp. NMC-1 | 1040 | Nostocales | 4854 | 168399.2163 | 34.6928 | 6.353 |
| *Trichormus variabilis* | 1040 | Nostocales | 5604 | 209432.1637 | 37.3719 | 6.434 |
| *Tychonema bourrellyi* | 1474 | Oscillatoriales | 4292 | 148847.6334 | 34.6802 | 6.275 |
| *Vulcanococcus limneticus* | NA | Synechococcales | 3392 | 108925.8833 | 32.1125 | 7.033 |
| *Westiellopsis prolifica* | 1590 | Nostocales | 5556 | 204443.4314 | 36.7968 | 6.529 |
| *Xenococcus* sp. PCC7305 | 1385 | [Pleurocapsales](https://www.ncbi.nlm.nih.gov/Taxonomy/Browser/wwwtax.cgi?mode=Undef&id=52604&lvl=3&lin=f&keep=1&srchmode=1&unlock) | 5057 | 183538.1155 | 36.2938 | 6.195 |
