# Supplementary material for: Virtual 2D map of cyanobacterial proteomes
Source: PLoS One. 2022 Oct 3;17(10):e0275148. doi: 10.1371/journal.pone.0275148 (PMC9529120; doi:10.1371/journal.pone.0275148)
Supplement: Attachment — Submitted filename: BDM_ Recommendation.docx [file pone.0275148.s010.docx]

The authors have addressed all the comments. However there are a very few grammatical errors should be addressed.

**I recommend this article for publication.**
