# Supplementary material for: Virtual 2D map of cyanobacterial proteomes
Source: PLoS One. 2022 Oct 3;17(10):e0275148. doi: 10.1371/journal.pone.0275148 (PMC9529120; doi:10.1371/journal.pone.0275148)
Supplement: Attachment — Submitted filename: Rebuttal_Response to reviewer comments.docx [file pone.0275148.s009.docx]

Editor and Reviewers

Many thanks for your time and constructive suggestions to improve the quality of the manuscript. We have accepted all of your suggestions and incorporated in the manuscript. For details, please find the track change manuscript with mark. We have also submitted a clean file of the track change file.

Reviewer #1: The manuscript submitted by Mohanta et al. has described the virtual 2D map of the cyanobacterial proteomes. They analyzed the Cyanobacterial proteomes of 229 species and reported the basic proteomics. The study contains each fundamental detail of the cyanobacterial proteome. It is promising research and holds high value get to publish in PLoS One. However, the article needs appropriate revision as per the comments provided below before acceptance for publication.
Overall comments; but the explanation should be reflected in the manuscript file to convey the importance of the study to the audience

Response

Thank you very much for your nice comments.

1. Why authors have included 229 species in their study? Do they have some commonalities or chosen as random?

Response

We have included all the Cyanobacterial proteomes available during the start of the analysis. When we started the analysis, there were 229 fully annotated cyanobacterial proteomes were there and hence we included all the species.

2. How the results of the cyanobacterial proteome can be corroborated with the bacterial or plant proteome. Please explain the similarities and differences, if any?

Response

So far, detailed study regarding the bacterial proteome is not available. However, we have explained the similarities and differences of Cyanobacterial proteomes and plant proteomes. In table 2, you can see the composition of cyanobacterial amino acids. The abundance (%) of Cys was the lowest in Cyanobacterial proteome whereas the Trp (1.28%) was the lowest abundance amino acid in plants. The abundance of Cys in plant kingdom was 1.85%. We added this in the text.

3. Is there any correlation with regard to molecular weight and isoelectric point of cyanobacterial proteomes?

Response: There is a negative correlation between molecular weight and isoelectric point of cyanobacterial proteins/proteome. Authors have already mentioned it in the manuscript. The correlation was -0.197.

4. Why Cys is lowest and Leu is highest abundant amino acid in the cyanobacterial proteomes. Although, it is difficult to establish the reason, do authors have any information or hypothesis behind this?

Response: As of now authors have no idea about the lowest abundance of Cys amino acids in cyanobacterial proteome.

5. What is the possible reason for the presence of higher average basic pI proteins (8.62) in cyanobacteria?

Response: The majorities of cyanobacterial species found in marine habitat. The pH of marine ecosystem is more than 8 and hence this might be the possibility that organism tried to have their pI level close to their environment/habitat.

Abstract:
I. Rewrite the sentence “…..The 20 genome sequence data of the Cyanobacteria has been extensively studied, but a detailed 21 proteomics study of the Cyanobacteria is lacking….” for better meaning and simplicity.

Response

There is nothing as such 20 or 21 mentioned in the text of the abstract. I presume, these number came during copying the sentence. Now we have revised the mentioned part.

II. Rewrite the sentence “…..The Cyanobacterial proteome encoded a 27 greater number of acidic-pI proteins, and the average pI of the Cyanobacterial proteome was found 28 6.437…..”.

Response

We have modified the sentence. Thanks for the suggestion.

III. Write either full name or its abbreviation any one “molecular weight and pI”

Response

We have provided the abbreviations for both the term and used them uniformly.

IV. Pls. rewrite “…Using this computational analysis…..”
Response: We have revised the mentioned part.

Introduction:
i. Make a single sentence “Therefore, proteomics provides more information about an organism compared to its genome. Proteomics provides an understanding of the cellular function and serves as a link between gene expression and translational products.”

Response

We have merged two sentences and made it as follows: The proteome provides more information than the genome, including an understanding of cellular function and the potential links between gene expression and translation.

ii. Rewrite the sentence “However, we lose lot of information about the whole proteome study as it is not possible to conduct 2D gel electrophoresis below pH 3 and above pH 11 due to lack of IPG (immobilized pH gradient) stripe below pH 3 and above pH 11.

Response

We have revised the sentence. Please find it. Thanks
iii. ………proteomic details can be written as ……..proteomics data…….

Response: We have revised the sentence. Please find it. Thanks

iv. Please rewrite this as both the consecutive sentences are started with Therefore….. line 81-86

Response

Thanks for the suggestion. We have revised it.

The introduction section is nicely presented but moderate English editing will be more helpful to the diverse audience to understand this informative work.

Response

The article is already edited by professional English editing Services “Journal doctors”. A certificate of English editing is appended at the end of the file. Further, we have rechecked it with Grammarly premium software and wordtune to remove any possible typos.

Results
I. The results part is well described as per the aim and objectives of the Manuscript by the authors; however, the pictorial presentation would be more updated by increasing the resolutions.

Response

Thanks for the suggestion. All the figures are more than 300 DPI. At the present they occupied a full page and hence get stretched. They will be look excellent in the printed/online version.

II. The author should also mention the image source and the software used to make the images.

Response: We have mentioned the image source in each Figure legend.

III. Units of the parameters should cross-checked.

Response

Thanks for the suggestion. We have cross-checked the units and modified if required.

Discussions
I. The authors have added sufficient discussion on their works and if within this time any more literature has been published, please add them in the discussion part which could give a complete frame for this work.
II. The introduction section is nicely presented but moderate English editing will be more helpful to the diverse audience to understand this informative work.

Response: Thanks for the nice suggestion. We have edited the manuscript from professional English editing services and further checked with wordtunes and Grammarly. Also, added a few more references to the existing discussion.


Conclusion
The author should add a paragraph on future prospects of these types of study in the application to the real scientific world. As this work is indeed a time taking analysis based on the data available in the database but it needs a very clear futuristic direction that can encourage following researchers.

Thanks for the suggestion. We have elaborated the suggested part.

Materials and Methods

I. In this section, the Author should add a table providing details on databases (online/offline) and other internet sources which will be essential to carry out such type of study. It would be helpful for young budding researchers to handle such big data sets.

Response: I think a step-wise presentation is better than a tabular format. We have presented the study in a step-wise manner and hence the current presentation will be a better way than presenting it in tabular form.

II. Authors should add abbreviations.

Response: We have mentioned the required abbreviations. As per journal criteria, first abbreviation can be followed throughout the manuscript. There is no specific abbreviation sections for PLoS One.


Moderate English language revision is a must prior to considering this manuscript for the publications.

Response

We have edited the MS for English literature.

Reviewer #2: - This study was designed similar to authors previous publication on ‘Virtual 2-D map of the fungal proteome’. This study used the sequences of 229 cyanobacterial species to analyse the molecular mass, average number of amino acids and isoelectric point of cyanobacterial proteome.

Response

You are correct find out our previous publications. We are trying to establish a kingdom-wide similarities and differences in proteomics and their amino acid composition. So that we can able to understand the evolutionary context of individual kingdom in the context of proteomics. Further, tRNA anticodons and CDS based codon study is under progress to establish the correlation. Once we finish the proteome, tRNA anticodon, and codon, it will be possible to answer the protein translation mechanism and their evolutionary consequences.

- This study focussed only on numbers and statistical data, and it lacks functional analysis.

Response: Yes, the study is more a computational and statistical analysis of proteomics. The aim of the study was not to conduct a functional analysis. Also, it is practically not feasible to conduct experimental study of 229 proteomes.

- Following suggestions may improve the quality of manuscript.
- The whole structure of the manuscript needs to be reorganised.
- The headings in the results section seems like long sentences rather than section headings. It should be descriptive and as concise as possible.

Response: we have modified the headings in a few parts and made it more concise.

- Table.1.: it can be provided as supplementary information. The information in this table can be segregated to identify the species with ascending or descending order of no of sequences/mol wt/pI.

Response: We have moved Table 1 as Supplementary Table. The table is already in Alphabetical order for the species name. There are multiple variations (e.g. sequence number, highest molecular weight, lowest molecular weight, highest pI, lowest pI etc.) in the data table. Therefore, it is good to organize the table according to the alphabet order.

The functional analysis should be discussed in the discussion section. For example, the authors should extract the information on species with industrial applications and its correlation to above parameters.

Response

Dear reviewer, many thanks for the nice suggestion. However, we have not conducted the study based on any particular protein or a group of proteins. Neither, the study is based on any particular species. The result is based on the whole analysis of 229 species. Further, the study is more focused towards genomics/codons and amino acid composition rather than industrial application. However, the highest and lowest amino acid composition based proteomes can be looked into for any possible research oriented or industrial application. We have already mentioned it in the text.

- Table.2. Significance of highest and lowest percentage of amino acids and its correlation to different species should be discussed in the text.

Response: Thanks for the suggestion. Now we have extended the discussion and mentioned it in the text. Reviewer can find about the P. marinus and V. limneticus about this.

- The manuscript should also focus the discussion on evolutionary time scale. Evolution of species and order may be represented in cladograms. Divergence and functional variations should also be discussed in the manuscript.

Response: In the previous submission, reviewer has asked to make it in tabular form for the evolutionary time scale. Although the report of evolutionary time scale with regard to the present proteomic study is not corroborative, still authors included it based on the suggestion of the reviewer and tried to corroborate it.

It might fit to make a cladogram. Because, we have used the individual protein sequences and for the analysis. To make any phylogenetic tree we need the protein sequences or a genome sequence. Here it is not possible to use more than 900K protein sequence to make a cladogram. Also, we can not use the genome file to make the cladogram and the study is based on proteome/protein sequences. Hence we can not use the genome sequences for the cladogram.

Author would like to mention that we have deduced the time scale using of individual species using Time tree server <http://www.timetree.org/>. We have mentioned it in the text and reader can easily navigate into it to resolve any confusion regarding cladogram and evolutionary history. If someone need to understand the timeline of evolution, it need to put the taxon name in the server to get details of evolutionary time scale and divergence. We have now mentioned it in the method section.

Authors have no idea how functional variation can be discussed in this study when no functional study was conducted. The study is basically based on protein sequences and proteomes.

- Majority of the conclusion section is almost hypothetical. This paper not concluded anything relevant to the functional and structural analysis.

Response: Authors have no idea for what purpose reviewer has mentioned about the functional or structural analysis. Indeed, its not a structural or functional study. It is a basic analysis of sequence information to get basic biological details of the cyanobacterial kingdom. These data can be valuable to understand the codon usage bias that play important role in gene expression and evolution.

- Figure 6. B was not explained anywhere in the text. Figure legend should contain the information on colour patterns.

Response: Now we have mentioned it in the text

- Figure 5 legend should be precise. Remove the analysis part in figure legend.

Response: We have removed the analysis part from the legend.

Reviewer #3: Decoding the Virtual 2D Map of Cyanobacterial Proteomes
The virtual 2D map of the cyanobacteria proteomes is reported in the manuscript. The authors reported the fundamental proteomics after analyzing the 229 species of cyanobacteria proteomes. Every essential aspect of the cyanobacteria proteome is covered in the study. The research is exciting and valuable enough to be published in PLoS One. Before being accepted for publication, the piece must, however, be appropriately revised in accordance with the remarks offered below.

Response: Thanks for the nice comments and finding it suitable for publication in PLoS One.

Comments:
1. The title of the manuscript should be modified.

Response: We have modified the title. We have deleted the “Decoding the” part from the title. Now the title is “Virtual 2D Map of Cyanobacterial Proteomes”
2. If possible graphical abstract should be provided.

Response: Thanks for the nice suggestion. However, PLoS One does not accept graphical abstract and hence we have not submitted a graphical abstract for this.
3. Line no 412-414: Protein sequences of 229 cyanobacteria species were downloaded from NCBI and proteomics details of these organisms were analyzed.

Response: I cannot see any question from the reviewer regarding this sentence.

4. The author should give a strong hypothesis before this study by taking huge data sets.

Response:
5. Why authors have considered 229 species and it will be better if the author would give a justification?

Response: When we started the analysis, the annotated protein sequences of 229 species were available. Therefore, authors have used 229 species in this study. We have mentioned this in the text.

6. Why author has taken the parameter of analysis like molecular weight and isoelectric point in this study?

Response: In proteomics, molecular weight and pI plays critical role in their subcellular localization and function. Further, in 2D proteomics, proteins are separated based on their molecular weight and isoelectric points. Therefore, authors have used the molecular weight and pI for the analysis.
7. Line no 418 and 419: For analysis, recent data should be taken into account if available.

Response: Thanks for the nice suggestion. We have collected all the annotated proteome sequences available till January 2021 and analyzed them. Genome sequence data are getting added every day. In the middle of the revision, it will not be wise to add any additional data. If we will add, the whole data need to reanalyze again. As the sequence data are getting added frequently, if we will follow this principle, we have to add the data in every revision and it will be difficult to publish any findings. Still, 229 proteomes is high enough to remove any statistical ambiguities as sample size is too high.

8. Line no 419 and 420: Only one proteome file was considered for 420 the analysis of those species contained repetitive protein sequence data. Is there any specific reason for that? If yes please mention it if possible.

Response: A proteome file contains multiple sequences. However, we have merged all the sequences and made a single file for individual species. These single files of individual species were used to identify the repetitive sequences. Looking into the confusion, we have deleted the sentences.

9. Line no 420 and 421: The data analysis starting date was already mentioned, there is no need for repetition.

Response: Thanks for the suggestion. We have mentioned it only once in the text.
10. Line 427 and 428: Please provide the script if possible.

Response: Thanks for the suggestion. Authors have not used any special scripts for the analysis. The script can be found in the program. Now we have mentioned the script in the text.

11. Line 437-439 and Line 446-447: Please explain how JASP Unscrambler 11, and time tree: the time scale of life server works.

Response: Now we have expanded the explanation of the mentioned software. Please find details in the method section.

12. In Fig 6 (b): Is there any particular explanation regarding different color coding between the interactions? If yes, please mention it.

Response: There is nothing as such legend available for the colors of Figure 6B. However, from the correlation plot of Figure 6A, we can notice that strong blue line is for the strong positive correlation. We have now mentioned it in the legend section.
13. All figures need a better resolution.

14. The author should also mention the image source and the software used to make the images.

Response: We have added the details about the source of the figure in each legend.

15. The author must add a paragraph on future prospects

Response: The conclusion section was with the future perspectives. Now we have modified the heading as “conclusion and future perspectives”.
