# Supplementary material for: Virtual 2D map of cyanobacterial proteomes
Source: PLoS One. 2022 Oct 3;17(10):e0275148. doi: 10.1371/journal.pone.0275148 (PMC9529120; doi:10.1371/journal.pone.0275148)
Supplement: Attachment — Submitted filename: Rebuttal_Response to reviewer comments.docx [file pone.0275148.s011.docx]

Dear

Editor and Reviewers

Many thanks for your time and constructive suggestions to improve the quality of the manuscript. We have accepted all of your suggestions and incorporated in the manuscript. For details, please find the track change manuscript with mark. We have also submitted a clean file of the track change file.

Reviewer 1.
The authors have revised the manuscript very well and now acceptable for publication. However, I have a few minor comments that need to be looked after for future.

Response: Many thanks for finding our article suitable for publication.

1. Do the correlation of pI and Molecular weight has any impact on structural aspects of the protein?

Response: The pI and molecular weight of protein does not have any impact on the structural aspect of the protein. However, it works in opposite. The structural aspect of the protein has role in the determining the pI and molecular aspects of the protein. We have mentioned it in line 280.

2. Can these Molecular weight and pI play role in structure of the protein?

Response: The question is similar to the previous one. We have mentioned it above. Molecular weight and pI do not play any role in structure of the protein. However, the structure of the protein decide the molecular weight and pI.

Reviewer 2.
I agree with the authours response to my queries, and recommend this manuscript for publication

Reviewer 3.
The authors have addressed all the comments. However there are a very few grammatical errors should be addressed. I recommend this article for publication.

Response

Thanks for the suggestion. We have checked for minor English typos.
